# Vestibular Stimulation on a Motion-Simulator Impacts on Mood States

**DOI:** 10.3389/fpsyg.2012.00499

**Published:** 2012-11-20

**Authors:** Lotta Winter, Tillmann H. C. Kruger, Jean Laurens, Harald Engler, Manfred Schedlowski, Dominik Straumann, M. Axel Wollmer

**Affiliations:** ^1^Division of Clinical Psychology and Sexual Medicine, Department of Psychiatry, Social Psychiatry and Psychotherapy, Hannover Medical SchoolHannover, Germany; ^2^Department of Neurology, Zurich University HospitalZurich, Switzerland^#^; ^3^Institute of Medical Psychology and Behavioral Immunobiology, University Hospital Essen, University of Duisburg-EssenEssen, Germany; ^4^Psychiatric Hospital of the University of BaselBasel, Switzerland

**Keywords:** vestibular system, motion-simulator, hexapod, mood states, cortisol, sensory perception

## Abstract

We are familiar with both pleasant and unpleasant psychotropic effects of movements associated with vestibular stimulation. However, there has been no attempt to scientifically explore the impact of different kinds of vestibular stimulation on mood states and biomarkers. A sample of 23 healthy volunteers were subjected to a random sequence of three different passive rotational (yaw, pitch, roll) and translational (heave, sway, surge) vestibular stimulation paradigms using a motion-simulator (hexapod). Mood states were measured by means of questionnaires and visual analog scales. In addition, saliva cortisol and α-amylase samples were taken. Compared to a subliminal control paradigm all rotational and two translational stimulations produced significant changes in mood states: Yaw rotation was associated with feeling more comfortable, pitch rotation with feeling more alert and energetic, and roll rotation with feeling less comfortable. Heave translation was associated with feeling more alert, less relaxed, and less comfortable and surge translation with feeling more alert. Biomarkers were not affected. In conclusion, we provide first experimental evidence that passive rotational and translational movements may influence mood states on a short-term basis and that the quality of these psychotropic effects may depend on the plane and axis of the respective movements.

## Introduction

Most people are familiar with the vivid interrelation between vestibular sensations and mood states. Vestibular sensations may be associated with pleasant or with unpleasant mood states. On the one hand there is an intense relation between vertigo and fear (Mazur and Booth, 1998; Best et al., 2009). On the other hand people often seek movements that are associated with vestibular stimulation to experience relaxation or euphoria. The need for these movements can be observed throughout life from newborns and infants in the cradle to the aged in a rocking chair. It may be most pronounced in childhood and underlie the affinity of children to swings and seesaws on playgrounds and to fairground rides. In adulthood commitment to sedentary professional occupation can lead to relative deprivation of vestibular stimulation, which may be compensated by sports and dancing.

In some cultures, rotatory movements are used to modulate mental experience. The whirling Dervishes of the Mawlawiyah order rotate around the longitudinal body axis to induce a state of religious ecstasy. Moreover, the “Five Tibetan Rites,” a series of exercises of unknown tradition that is supposed to promote relaxation and well-being, comprise one exercise of clockwise spinning around the longitudinal body axis (Kelder, 1939). Predominantly in the early nineteenth century, high-speed rotation in “Cox’s chair” was used in psychiatry to break states of psychomotor agitation (Wade et al., 2005). According to Dr. Cox’s description a chair was suspended from the ceiling by means of ropes. Subjects were seated securely in this chair and rotated at a given not further specified speed by an attendant around a vertical (or longitudinal) axis for a given period of time. Usually, it was used to induce sleep in mania. There are anecdotal reports that patients used the device voluntarily to attain positive effects on mood by repeated application. In demented patients, swinging has been suggested as an intervention to improve relaxation and emotional well-being (Snyder et al., 2001; Wade, 2005; Kelly, 2008; Breathnach, 2010).

From a clinical point of view there is a strong coincidence between vertigo and mental disorders, such as depression or anxiety disorders, particularly acrophobia or agoraphobia (Pollak et al., 2003; Soza Ried and Aviles, 2007; Best et al., 2009). Moreover, mood influences the ability to keep one’s balance (Bolmont et al., 2002) and side asymmetry in the activity of the vestibular nuclei has been observed in depressed patients (Soza Ried and Aviles, 2007). Conversely, dysfunction of the vestibular system may trigger anxiety and symptoms of depression. Improvement of accompanying mental symptoms has been observed during vestibular rehabilitation (Yardley et al., 1998) and selective serotonin reuptake inhibitors (SSRI) may be beneficial in the treatment of vertigo (Staab et al., 2002).

The neurobiological correlates of these phenomenological interrelations may be projections from vestibular nuclei to cortical and sub-cortical brain regions that are also involved in the regulation of mood states (Jacob and Furman, 2001). These regions include the insula, the cingulate, the hippocampus, and the parabrachial nucleus. Conversely, the dorsal raphe and the locus coeruleus, two important structures in the regulation of mood states, send out serotonergic and noradrenergic projections to vestibular nuclei in the brain stem.

We hypothesized that movements associated with vestibular stimulation may produce differential mood effects depending on their planes and axes. To test this hypothesis we measured mood states of healthy subjects during a random sequence of rotational and translational vestibular stimulation paradigms on a hexapod motion-simulator. Biomarkers such as saliva α-amylase and cortisol were assessed to detect changes of activity of the hypothalamus-pituitary-adrenal axis (HPA axis) and the sympathetic nervous system.

## Materials and Methods

### Subjects

Twenty-three healthy volunteers (mean age of 31.1 ± 8.5, SE; range 22–59 years) participated in this study after providing written informed consent. Ten of them were male (mean age 35.1 ± 10.7; range 25–59) and 13 were female (28.0 ± 4.9; range 22–38). The subjects were recruited via advertisement on the internet and on notice-boards at the University of Zurich, Switzerland. The advertisements included the information that the relationship between the equilibrium sense and mood states was to be investigated. The study was approved by the local ethics committee.

All participants were screened by a semi-structured interview and by filling out a general socio-demographic and medical questionnaire, which enabled us to exclude individuals involved in drug/alcohol abuse, and those taking any kind of medication or suffering from any somatic or mental disorders. This questionnaire also included an assessment of current or previous disorders of the vestibular system using the main items of the German version of the Vertigo Symptom Scale (VSS-D; Yardley et al., 1992; Tschan et al., 2008). Subjects with any signs of such disorders were excluded from participation.

Since the assessment of mood states was a principal aim of the current study, subjects were additionally asked to complete the Beck Depression Inventory (BDI; Beck et al., 1961) to identify subjects with signs of a clinical depression. To exclude the fact that a propensity for sensation-seeking behavior might confound the rating of vestibular stimulation paradigms, we screened all participants with the Sensation-Seeking Scale (SSS; Kolin et al., 1964; Zuckerman and Link, 1968). None of the participating subjects had either a clinical score in the BDI or an elevated score in the SSS. During recruitment two subjects were excluded due to signs of a clinical depression.

### Design and procedure

A single-blind cross-over design was used incorporating an experimental and a control condition in randomized order. Each subject had to keep three appointments. The first one contained the examination of the criteria for inclusion and exclusion as well as a test stimulation for the subject to get used to the apparatus. The second and third appointments comprised the actual investigation sessions. At one of them the subject received six control stimulations, at the other one six experimental stimulations.

**Figure 1:**
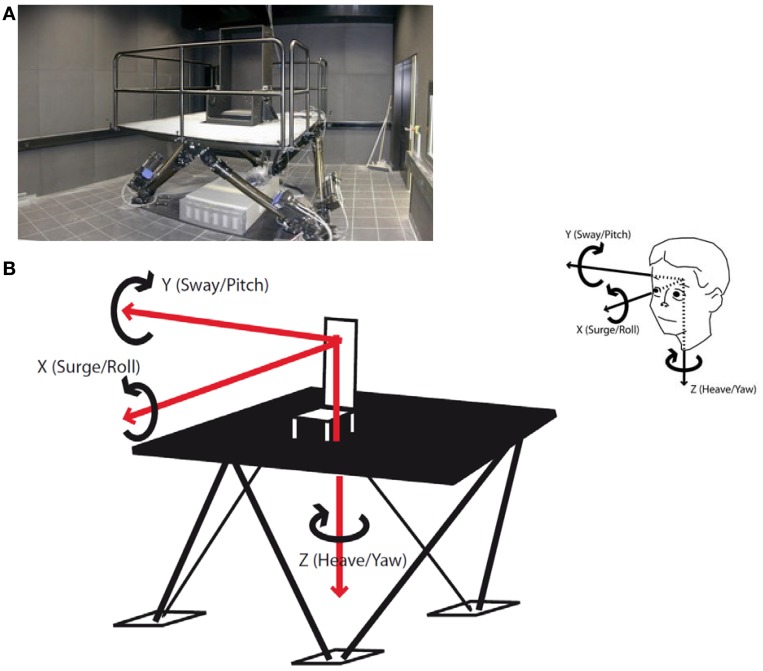
**(A)** The hexapod. A hexapod or Stewart platform is a mechanical device with six jacks mounted on a level surface in three pairs, crossing over to three mounting points on a top plate. Devices placed on the top plate can be moved in the 6-degrees-of-freedom in which it is possible for a freely suspended body to move. **(B)** Six forms of vestibular stimulation on the hexapod. The hexapod allows three linear movements which are depicted as red arrows: back–forth (*x*-axis, surge), left–right (*y*-axis, sway), and up–down (*z*-axis, heave). Additionally, the hexapod allows three rotational movements which are depicted as black loops: sagittal axis (*x*-axis, roll), cross axis (*y*-axis, pitch), and longitudinal axis (*z*-axis, yaw). All possible forms of motion are additionally depicted as projections on a head in the upper right corner of the figure.

The vestibular stimulation was performed on a hexapod (Stewart platform, Figure 1A), a mechanical device with six jacks mounted on a level surface in three pairs, crossing over to three mounting points on a top plate. Objects placed on the top plate can be moved with six, i.e., three rotational and three translational, degrees of freedom. The hexapod is a well-established tool in neurovestibular research. A hexapod allows highly accurate and computer-controlled separate stimulation of the six different sensory qualities of the human vestibular organ. Specifically, we used the E-Cue 624 × 18006-degrees-of-freedom motion platform (FCS Control Systems B. V, Netherlands, 2002).

**Figure 2:**
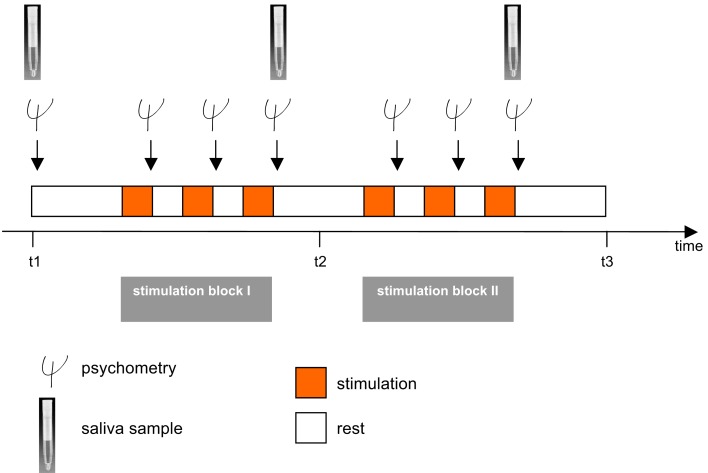
**The experimental paradigm**. The investigation consisted of two sessions including an experimental and a control/sham condition. In a cross-over design subjects were randomly assigned to both conditions. A session included two stimulation blocks each lasting 540 s, one with three rotational stimulations (yaw, pitch, roll) and one with translational vestibular stimulations (surge, heave, sway) which were performed on a hexapod. Translational and rotational stimulation blocks were also randomized, as were all forms of vestibular stimulation within the rotational and translational stimulation blocks. After each stimulation (100 s), there was a break of 120 s during which subjects had to immediately estimate their mood states. Between the two stimulation blocks there was a break of 300 s. Psychometric assessment was conducted before and after each vestibular stimulation. Saliva samples for assessment of cortisol and α-amylase were administered before (*t*1), between the two stimulation blocks (*t*2), and after the entire stimulation (*t*3). A detailed description is given in the methods section.

Participants were seated in a chair mounted on the platform and were secured with a 4-point safety harness. Postural stability was ensured by a headrest and a horizontal bar which subjects held onto. Stimulations took place in complete darkness to preclude visual adjustment of vestibular stimuli. Stimulations were classified into translations, i.e., linear movements in three cardinal directions: back–forth (*x*-axis; surge), left–right (*y*-axis; sway), and up–down (*z*-axis; heave; Figure 1B) and rotations, i.e., circular motions in the three cardinal directions: sagittal axis (roll), cross axis (pitch), and longitudinal axis (yaw). The frequency in all six stimulations was 0.25 Hz. The translations were run with an amplitude of 35 cm (i.e., peak to peak displacement of 70 cm) and the rotations with one of 12° (i.e., peak to peak displacement of 24°) with each stimulation lasting 100 s. Between two stimulations there was a break of 120 s which was used to collect the psychometric data (Figure 2). It also allowed the vestibular system to rest before the next stimulation started. Stimulations were conducted in two blocks each lasting 540 s in total. One block comprised the rotational stimulations, the other comprised the translational stimulations. Between the translational and the rotational block there was a break of 300 s. Also at the beginning and at the end of the paradigm there was a block of 300 s. Thus, the entire stimulation paradigm on the hexapod lasted about 33 min. Control conditions consisted of subliminal stimulations. For that purpose frequencies were minimized, while the amplitude in the translations and the angle in the rotations remained unaltered. The resulting very slow movements of the hexapod were adjusted to the inertia of the vestibular organ and were below its detection threshold. However, the level of noise production of the hexapod was similar in each session, so that subjects were not able to distinguish between control and experimental session immediately. The order of stimulation and control sessions, of rotation and translation blocks, and of the three paradigms within the respective blocks was randomized. To minimize possible effects of circadian rhythms on variance in mood states and biomarker measurements, all sessions took place between 2 p.m. and 8 p.m., with both control and experimental sessions for each subject taking place at the same time.

### Measures

#### Psychometric measures

For repeated measurements of mood states we used five 100 mm visual analog rating scales (VAS) and the German short version of the Multidimensional Mood State Questionnaire (Mehrdimensionaler Befindlichkeitsfragebogen, MDBF; Steyer et al., 1997). VAS are sensitive and valid tools for the immediate assessment of mood and psychophysiological alterations (Zealley and Aitken, 1969; Kruger et al., 2003). The five VAS were “good–bad,” “energized–exhausted,” “relaxed–tense,” “comfortable–uncomfortable,” and “alert–sleepy.” The MDBF is a self-reporting instrument to measure the current psychological and mood state in three global dimensions (“good mood–bad mood,” “alertness–tiredness,” and “calmness–agitation”). After each stimulation participants were asked to rate how they felt during the stimulation by completing the five VAS as well as the MDBF (Figure 2).

For a more comprehensive assessment of mood states, each subject completed the Profile of Mood States Bipolar (POMS-Bi; Lorr et al., 2003), a questionnaire with 72 items which sum up to the six scales “composed-anxious,” “agreeable-hostile,” “elated-depressed,” “confident-unsure,” “energetic-tired,” and “clearheaded-confused,” three times during each session. Once before the stimulation started, a second time between the two stimulation blocks, and a third time after all stimulations were accomplished (Figure 2).

We also asked participants for brief free text comments and descriptions of associations they had during stimulation, as well as after each stimulation paradigm.

#### Biomarkers

To detect possible stress responses during the experiment, we collected saliva samples using Salivette collection devices (Sarstedt, Rommelsdorf, Germany) and measured salivary cortisol levels and α-amylase activity as markers of the HPA axis and sympathetic nervous system activation respectively (Kirschbaum and Hellhammer, 1994). In analogy to the POMS-Bipolar questionnaire, saliva samples were taken before stimulation (*t*1), between the two stimulation blocks (*t*2), and a third time after stimulations (*t*3). Samples were centrifuged at 1000 × *g* for 2 min and then stored at −20°C.

Salivary cortisol levels were measured using a commercial enzyme-linked immunosorbent assay (Cortisol ELISA, IBL International, Hamburg, Germany) according to the manufacturer’s instructions. Cross-reactivity of the anti-cortisol antibody with other relevant steroids was 7.0% (11-deoxycortisol), 4.2% (cortisone), 1.4% (corticosterone), 0.35% (progesterone), and <0.01% (testosterone, estrone, estradiol, estriol). Intra- and interassay variances were 4.8 and 5.9% respectively.

Salivary α-amylase activity was determined using a commercially available enzymatic assay (Salivary α-Amylase Assay Kit, Salimetrics, State College, PA, USA) according to the manufacturer’s instructions. Briefly, diluted saliva (1:200) was mixed with a prewarmed (37°C) solution of 2-chloro-*p*-nitrophenol linked to maltotriose. The enzymatic conversion of this substrate by α-amylase yields 2-chloro-*p*-nitrophenol, which can be spectrophotometrically measured at 405 nm. The increase in absorbance at 405 nm over a period of 2 min is directly proportional to the amount of α-amylase activity present in the sample. Intra- and interassay variances were 2.4 and 3.5% respectively.

### Statistical analysis

As normal distributions could not be proven for all psychometric data, the non-parametric Wilcoxon Test was used to compare medians. For the biomarker measures normal distribution could be verified. These data were analyzed by a two-way analysis of variance (ANOVA, condition × time) for repeated measures. Data were analyzed using PASW 18.0 (SPSS Inc., Chicago, IL, USA). They are presented as mean ± S.E. Statistical significance was assumed if raw *p* values were ≤0.05 for all analyses.

## Results

### Psychometric measures

**Figure 3:**
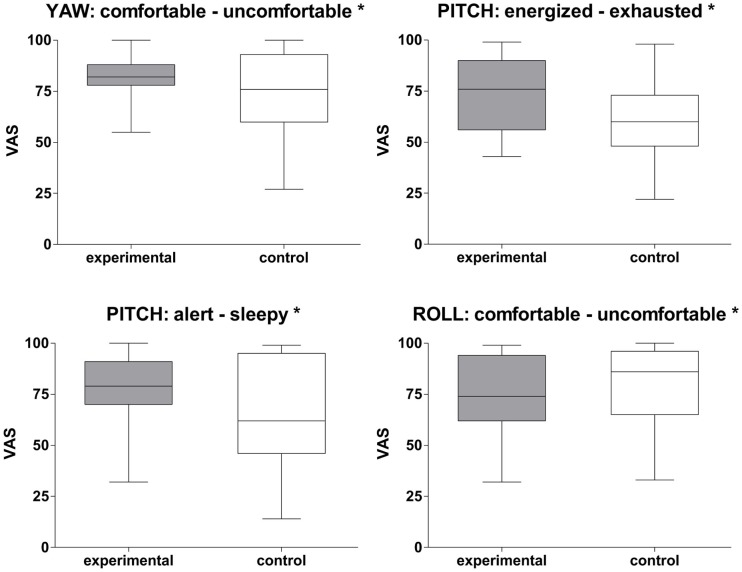
**Rotational stimulation and impact on mood states**. Box plots of significant effects of the three rotational stimulations on mood states using visual analog scales (VAS) in the control and experimental condition. Box plots showing the median, upper and lower quartile, with whiskers showing the minimum and maximum of all values. High values on the VAS indicated that the positive adjective of the pair was appropriate and vice versa for low values. *p < 0.05.

All three rotational stimulation paradigms were associated with specific alterations in mood states as measured with the VAS. In comparison with the control condition, subjects felt significantly more comfortable during the yaw rotation (*Z* = −2.14, *p* < 0.05; Figure 3). Being rocked in a cradle or by a boat on the sea was frequently quoted as a comparable experience. The pitch rotation induced a state of feeling more energized (*Z* = −2.50, *p* < 0.05) and alert (*Z* = −2.16, *p* < 0.05) than the control stimulation. Almost all participants reported that it reminded them of swinging on a swing or in a swing-boat. In contrast, the roll rotation induced a state of feeling less comfortable (*Z* = −2.14, *p* < 0.05). This was associated with, for instance, being on a boat on a rough sea or in a cable car during a storm.

**Figure 4:**
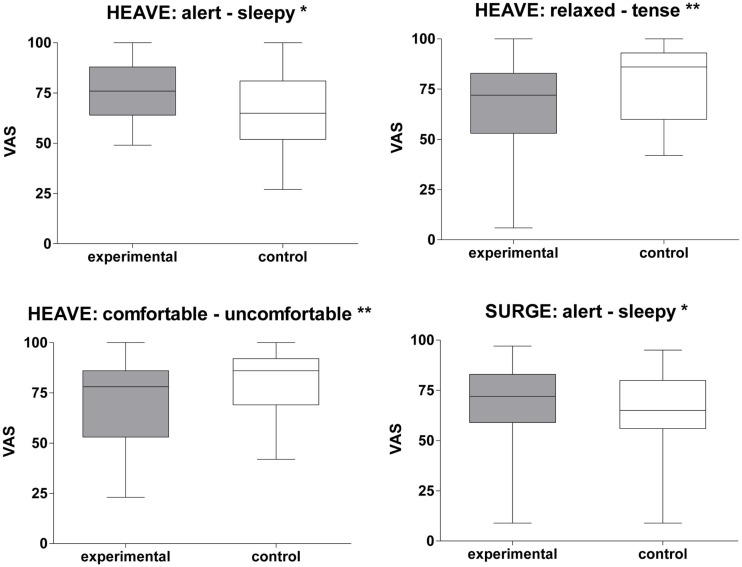
**Translational stimulation and impact on mood states**. Box plots of significant effects of two translational stimulations (surge and heave) on mood states using VAS scales in the control and experimental condition. Box plots showing the median, upper, and lower quartile, with whiskers showing the minimum and maximum of all values. High values on the VAS indicated that the positive adjective of the pair was appropriate and vice versa for low values. *p < 0.05; **p < 0.01.

Among the three translational stimuli, the heave translation induced the most prominent effects on mood states. As measured by VAS scores participants felt more alert (*Z* = −2.42, *p* < 0.05), less relaxed (*Z* = −3.25, *p* < 0.01), and less comfortable (*Z* = −2.92, *p* < 0.01) during this stimulation than under control conditions (Figure 4). Accordingly, the heave paradigm was associated with higher levels of agitation on the MDBF (*Z* = −2.08, *p* < 0.05). Most subjects reported associations like free fall, being in a lift, or jumping on a trampoline. Similarly, the surge stimulation paradigm induced a state of greater alertness (*Z* = −1.96, *p* < 0.05) than the control stimulation did. The most common association was being in a car during stop-and-go traffic. In contrast, the sway translation did not show any effects on mood states as measured by either VAS or MDBF. Associations with this movement were diffuse and it was assessed as the most unusual or most foreign compared to everyday movements.

The scores of the POMS-Bipolar questionnaire revealed block-wise alterations in mood states. After the first block of stimulations participants tended to feel tired (*Z* = −2.00, *p* = 0.045) and confused (*Z* = −2.18, *p* = 0.030). When the translational and rotational stimulation blocks were evaluated separately, subjects reported being more confused after translation (*Z* = −2.37, *p* = 0.018) and being more tired after rotation (*Z* = −2.34, *p* = 0.019). However, this effect was observed only after the first block of stimulations.

### Biomarkers

There were no biological signs of a stress response during the experiments. Translational and rotational vestibular stimulation did not specifically affect saliva levels of cortisol and α-amylase compared to control conditions. However, mean cortisol values slightly increased toward time point 2 (*t*2) with a subsequent decline toward tim*E*.e point 3 (*t*3) in both groups [mean values ± SD (SE) in nmol/l for control condition: *t*1 = 5.43 ± 0.64; *t*2 = 6.18 ± 0.95; *t*3 = 5.97 ± 1.22; and experimental condition: *t*1 = 4.48 ± 0.6; *t*2 = 4.73 ± 0.55; *t*3 = 3.91 ± 0.54], showing a significant time effect in the ANOVA analysis only for the experimental condition [*F*(2, 44) = 4.07, *p* = 0.015]. *Post hoc* analysis revealed a significant decline between *t*2 and *t*3 (*Z* = −2.85, *p* = 0.004).

Saliva levels of α-amylase slightly decreased toward the end of stimulation in both groups (mean values in U/ml for control condition: *t*1 = 189.37 ± 31.04; *t*2 = 179.31 ± 25.40; *t*3 = 171.97 ± 25.36; for the experimental condition: *t*1 = 199.61 ± 29.43; *t*2 = 200.42 ± 29.01, *t*3 = 169.24 ± 22.31). However, this effect was not statistically significant.

## Discussion

Despite the obvious interrelation of vestibular sensations and mood states, no controlled trials systematically investigating the impact of different vestibular stimulations on mood states and psychophysiological parameters have been published. Here we present the first study that describes differential affective responses to moderate, short-term, passive movement on a hexapod with 6-degrees-of-freedom as an experimental paradigm of vestibular-affective interaction in healthy humans.

Our experiments show an immediate impact of passive movements on the hexapod on mood states. Rotational and translational movements induce different mood states. A set of three different rotational movements induced a feeling of tiredness. A set of three different translational movements induced a feeling of confusion. For the rotational movements, the rotation axis had an impact on the reported mood states. Yaw rotation was associated with feeling comfortable, while pitch rotation was associated with feeling alert and energetic. Accordingly, for the translational movements the frontal plane had an influence on mood states. The heave translation was associated with feeling alert, less relaxed, and less comfortable. Surge translation was only associated with feeling alert and sway translation was affectively inert. Interestingly, the reported mood states were in line with everyday situations participants associated with the respective stimulation. For example the comfortable feeling induced by yaw rotation reminded subjects of being rocked in a cradle, which is used to pacify infants. The increased alertness after surge and heave stimulation may correspond to a psychophysiological startle response in potentially dangerous situations like falling, which was frequently associated by the participants.

Comparing rotational stimulations with translational ones, the former tend to induce relaxation and pleasure while the latter tend to promote alertness and tension. However, this does not apply to movements on the frontal plane, i.e., roll and sway movements. This again is comparable to situations in daily life where accelerations and decelerations (such as running, driving cars, stop, and go traffic) require a high degree of attention and physical preparedness, whereas rotational stimulations are mostly found in pleasurable situations. Early observations in the nineteenth century by Erasmus Darwin, Joseph Mason Cox, and William Hallaran support this notion by stating that rotational stimulation on the so-called Cox’s chair or Hallaran’s swing was enjoyed by some of their patients and has been used as mode of amusement (reviewed by Kelly, 2008; Breathnach, 2010).

The study shows immediate effects on mood states as assessed by visual analog rating scales, whereas other psychometric instruments, such as the MDBF or the POMS-Bipolar, depicted less pronounced alterations in mood states. This may indicate that brief instruments, such as visual analog rating scales, might be more useful in assessing short-term effects on mood. This assumption receives support from previous psychophysiological studies (Zealley and Aitken, 1969; Kruger et al., 2003). Moreover, it is possible that the induced mood effects were too fugacious or compensating to be recorded in the more detailed POMS questionnaire after a stimulation block. However, the study was designed for the systematical assessment of all six stimulation forms of the vestibular organ within one experiment. More pronounced and stable effects may occur after one specific vestibular stimulation with higher intensity (frequency or amplitude), longer duration, or several repetitions.

Such kind of stimulations could also be tested for clinical application, for instance, as an adjunctive treatment of depression and anxiety disorders. Indeed, although incorporating only a small sample, Soza Ried and Aviles (2007) found that the vestibular system of people suffering from depression is functionally affected in the sense that they have a hypoactive right vestibular nucleus. Interestingly, not only depressed subjects display reduced hippocampus volume size (McKinnon et al., 2009), patients with vestibular dysfunction also do so (Brandt et al., 2005; Smith et al., 2005) and this leads to disturbed cognitive function in both groups. Physiotherapeutic vestibular rehabilitation has been reported to alleviate symptoms of anxiety and depression (Whitney et al., 2005; Meli et al., 2007).

Although not examined in this study a brief look onto possible age differences in perceiving vestibular stimulation as pleasant or aversive might be interesting. We already assumed that the majority of children enjoys being spun around and shows positive emotional signs whereas adults may show both positive and negative reactions to similar stimulations, e.g., when experiencing fairground rides with some of them even avoiding such situations. In the current experiment none of the participants indicated any signs of dizziness, nausea, or other disturbances. However, the intensity of vestibular stimulation in this pilot study was chosen rather moderate and there might have been a bias toward subjects having an unproblematic relation to vestibular stimulation. It seems that adults may seek less intense vestibular stimulation such as dancing or doing other kinds of sports, though empirical evidence for such an assumption is completely lacking. However, when looking at motion sickness we see that the susceptibility for motion sickness begins from around 6 to 7 years of age and is peaking around 9–10 years (Golding, 2006a,b; Reavley et al., 2006). Golding (2006b) states that the reason for this is uncertain, but that one possibility is, that the perceptuomotor map is still highly plastic and not fully formed until around 7 years of age. Theories about the development of perception skills say that until the age of seven a psychophysiological learning process takes place and differentiates perception more and more (Fock, 2004). Thus, one might speculate that children seek intense vestibular stimulation before this age whereas afterward there is an increase of susceptibility for motion induced disturbances.

Finally, some methodological issues and limitations of the study should be addressed: The axis and plane specificity of the effects on mood states indicates that they are probably mediated by the vestibular organ. However, it cannot be excluded that other aspects of passive movement, like the viscero-afferent signal induced by acceleration and deceleration of inner organs, may contribute to altering mood states. Moreover, we cannot exclude that mood effects may be mediated by the affective connotation of the situations of which participants were reminded during stimulation rather than by the stimulation itself. This is an essentially descriptive study covering a broad variety of stimulation paradigms and using a small sample size, which may limit statistical power.

In summary, this pilot study provides preliminary experimental evidence that movements associated with vestibular stimulation may have plane- and axis-dependent effects on mood states. It may contribute to the understanding of the interrelation between the vestibular system and emotional experience and may be of potential clinical use in affective disorders. Apart from this, studies on the impact of sports on mental health (particularly states of depression) might consider to control the degree of vestibular stimulation. Finally, these results provide first evidence why and how certain everyday activities such as running, dancing, swinging, falling aside, or driving cars may exert positive and negative effects on subjective well-being and mood.

## Conflict of Interest Statement

The authors declare that the research was conducted in the absence of any commercial or financial relationships that could be construed as a potential conflict of interest.
